# Effect on haemostasis of different replacement fluids during therapeutic plasma exchange—A comparative multicentre observational study

**DOI:** 10.1002/jca.22008

**Published:** 2022-08-24

**Authors:** Ruchika Kohli, Elisa Allen, Sean Platton, James Griffin, Lynn Manson, Peter MacCallum, Laura Green

**Affiliations:** ^1^ Wolfson Institute of Population Health Queen Mary University of London London UK; ^2^ NHS Blood and Transplant Bristol UK; ^3^ Barts Health NHS Trust London UK; ^4^ University Hospitals Bristol Bristol UK; ^5^ Scottish National Blood Transfusion Service Edinburgh Scotland UK; ^6^ Blizard Institute, Queen Mary University of London London UK; ^7^ NHS Blood and Transplant London UK

**Keywords:** fluid replacement, haemostasis, therapeutic plasma exchange, thrombin generation

## Abstract

**Introduction:**

Therapeutic plasma exchange (TPE) is used for several chronic conditions with little evidence on the efficacy and safety of different choice of replacement fluid. Measurement of haemostasis, particularly in vitro thrombin generation, could play a role in determining the immediate efficacy of different fluid replacement.

**Aim:**

To determine the impact of different TPE replacement fluid regimens on haemostatic assays.

**Methods:**

Prospective observational multi‐centre cohort study in adult patients 18 years and older evaluating haemostatic changes between four different TPE regimens: (1) 5% human albumin solution (Alb) only, (2) 50:50 mix of 5% Alb + modified gelatin, (3) 70:30 mix of 5% Alb and normal saline (NS), and (4) solvent‐detergent, virus‐inactivated fresh frozen plasma (FFP) (either alone or combined with other fluids). Twenty‐one haemostasis variables were analysed (procoagulant, anticoagulant and fibrinolytic factors) pre and post TPE sessions, including in vitro thrombin generation. Linear mixed modelling and canonical discriminant analyses were used to examine the effect of TPE fluid type on haemostatic variables.

**Results:**

A total of 31 patients with up to 5 TPE sessions each (131 sessions in total) were enrolled. Out of 21 markers analysed using linear mixed modelling, the main effects of fluid type were found to be significant for 19 markers (*P* < 0.05), excluding plasminogen activator inhibitor‐1 antigen and thrombin‐anti‐thrombin. Multivariate Analysis of Variance showed significant differences between the fluid types (Wilks' lambda = 0.07; *F*_63,245.61_ = 5.50; *P* < 0.0001) and this was supported by a canonical discriminant analysis, which identified the 4 most discriminating markers for fluid types as thrombin generation (lag‐time, time‐to Peak), fibrinogen and Factor V. In our analyses, the effect of FFP on haemostasis was significantly greater compared with other fluid types. Of the non‐FFP fluids, 5% Alb + NS had a lower effect on haemostasis compared to other fluid types (Alb and modified gelatin + 5% Alb).

**Conclusion:**

Thrombin generation and fibrinogen discriminated better the effect of different TPE fluids on haemostasis and should be considered as potential markers to evaluate the immediate haemostatic effect of TPE procedures. The use of NS as a TPE replacement fluid had a distinctive impact on thrombin generation and fibrinogen responses compared to other non‐FFP fluids.

## 1 INTRODUCTION

Therapeutic plasma exchange (TPE) is used as a first line of treatment for several chronic conditions to remove targeted circulating plasma antibodies, immune complexes or paraproteins and replace these with fluid such as plasma, colloid, crystalloid or a combination of fluids.^1^ The evidence upon which fluid replacement to use during TPE for different conditions, is lacking.^2^ In the UK, several options exist as a fluid replacement for TPE, such as electrolyte solutions (0.9% NaCl or Ringer lactate), human albumin solution (Alb), fresh frozen plasma (FFP), or a combination of these.^3^ The main factors influencing selection of each type of fluid are patient's bleeding risks,^4^ baseline albumin levels,^4^ availability of fluid,^5^ and cost of fluid.^2^

Each replacement fluid has advantages and disadvantages. There have been very few randomised controlled trials (RCT) to compare the efficacy and safety of different replacement fluids.^2^ One of the main reasons for the lack of evidence in this setting, is the absence of consensus on which outcome measures to use to determine the clinical efficacy of different fluids during TPE, particularly as TPE is most commonly used for management of chronic conditions (like Myasthenia gravis, Goodpasture syndrome etc.), meaning that any trial using a disease‐specific outcome would be lengthy, costly, and complex to run.

In addition to removing the targeted abnormal plasma proteins, TPE also removes normal plasma proteins with resultant disturbance of the haemostatic balance, which potentially could increase the risks of bleeding or thrombosis. Biological markers of haemostasis could be used to determine the immediate effect of different replacement fluids, and thus they can potentially be used as outcomes to evaluate the short‐term efficacy of TPE. Indeed, studies that have examined the impact of TPE on haemostasis have shown a significant immediate decline in fibrinogen and other coagulation factors after plasma exchange, with the recovery of these factors occurring at 24 to 72 hours later.^6^, ^7^, ^8^, ^9^, ^10^ However, the usefulness of standard coagulation tests is particularly limited with respect to the detection of hyper‐ or hypo‐coagulable states. Over the last few decades the use of global clotting tests, like in vitro thrombin generation test (TG), have shown promising results in exhibiting haemostatic capacity of individuals with acquired or inherited thrombophilic defects,^11^ bleeding disorders^12^, ^13^ or patients undergoing surgery.^14^, ^15^ To our knowledge, no studies have compared the effect of different types of fluid during TPE on the in vitro TG test, and in this paper, we aimed to do just that. Further, to complement this, we have also compared the impact of different replacement fluid regimens used for TPE on other haemostatic tests such as conventional clotting tests and clotting factors.

## 2 METHODS

This was a prospective multi‐centre cohort study in adult patients (≥18 years) undergoing TPE at three hospitals (two in England and one in Scotland). The study received research ethical committee (REC) approvals from Health Research Authorities in England (Integrated Research Application System number 247935) and Scotland (REC reference 18/LO/1954). All patients undergoing elective TPE procedures at each study site who were able to give consent were approached and asked for research blood samples only. The standard care of patients was not altered during this study. Prior to enrolment, written informed consent was obtained from all patients. Patients who underwent TPE treatment for thrombotic thrombocytopenic purpura, atypical haemolytic uraemic syndrome and related thrombotic microangiopathies were not included in this study, as TPE is a well‐established treatment in these patients.

### 2.1 Therapeutic Plasma Exchange procedure

Each hospital used a different replacement fluid regimen: Hospital‐1 used exclusively 5% albumin solution (Alb), Hospital‐2 used a 50:50 mix of 5% Alb and Gelofusine (succinylated gelatin or modified fluid gelatin^16^) as well as 5% Alb only, and Hospital‐3 used exclusively a 70:30 mix of 5% Alb and normal saline (NS). For patients who were deemed to be at high risk of bleeding (determined based on patient's clinical/family history, liver function tests and conventional coagulation tests, particularly fibrinogen), all sites used thawed plasma (Octaplas, a solvent‐detergent, virus‐inactivated FFP manufactured by Octapharma Ltd^17^) as the main replacement fluid (dose of Octaplas used was dependent on fibrinogen level, ie, if fibrinogen level was 1‐1.5 g/L, 0.5 L of Octaplas was administered, if fibrinogen level was <1 g/L, 1 L of Octaplas was administered). The Octaplas group was treated as a separate group in our analysis.

The TPE procedures at all three sites were similar. Standard of care applies at all hospitals and there is no evidence that everything else remaining equal, hospital influences haemostasis responses. The machines used for exchange were the COBE Spectra and Spectra Optia (Terumo BCT, Lakewood, CO), which use a continuous flow centrifugation‐based plasma separator technique. Each patient underwent 1‐1.5 plasma volume (PV) exchanges per session, calculated using the formula shown in below.

Eq. 1 Estimated plasma volume (EPV) calculation

$$\mathrm{EPV}\text{(in litres)}=0.07\;\times \;{\text{patient}}^{'}s\;\text{weight}\left(\mathrm{kg}\right)\times \left(1-\text{Haematocrit}\right).$$

*Result multiplied by 1.5 for 1.5 PV exchange*.

### 2.2 Blood sample collection and processing.

Research venous blood samples were collected immediately before and after each exchange procedure for up to five exchange sessions in each patient. Blood samples were collected in 5 mL BD vacutainers (BD Diagnostics, Oxford, UK) each containing 0.5 mL of 0.109 M buffered tri‐sodium citrate in a ratio of 1‐part anticoagulant to 9‐parts blood. Samples were processed within 4 hours of collection by double centrifugation at 2000*g* for 12 minutes to prepare platelet poor plasma (PPP) which was stored in aliquots at −70°C ± 10°C until day of processing.

### 2.3 Laboratory tests

Haemoglobin, platelet count, creatinine and albumin were analysed on each site as per laboratory standard operating procedures (SOP) using automated Sysmex XN analysers and the spectrophotometry, Roche Cobas 8000 analysers, respectively. All haemostatic tests were performed in one laboratory, and these included: prothrombin time (PT), activated partial thromboplastin time (APTT), fibrinogen, factor (F) II, FV, FVII, FVIII, FIX, FX FXI, FXII, alpha‐2‐antiplasmin (A2AP), plasminogen, prothrombin fragment 1 + 2 (F1 + 2), thrombin antithrombin complexes (TAT), plasminogen activator inhibitor‐1 antigen (PAI‐1) and thrombin generation test.

One‐stage clotting factor assays for FII, FV, FVII, FVIII, FIX, FX, FXI and FXII were performed on a Sysmex CS5100 analyser, using the laboratory's SOP and Siemens reagents. Enzyme linked immunosorbent assay (ELISA) were used for F1 + 2, TAT (using Siemens Enzygnost ELISA kits), A2AP, plasminogen assays and PAI‐1 (using Hyphen BioMed ELISA kit) following manufacturer's instructions.

### 2.4 Thrombin generation

The calibrated automated thrombogram method as described by Hemker et al.^11^ was used to perform the in vitro thrombin generation assay. Manufacturer's PPP reagents, which gave a reaction concentration of 5 pM for tissue factor and 4 mM phospholipid (Thrombinoscope BV), were used. The pre‐ and post‐TPE samples for each patient were tested at the same time to avoid inter‐assay variation. The following parameters were measured: lag‐time; the time to reach the peak (or ttPeak); peak‐thrombin height; velocity index (VI) (defined as = peak thrombin height/(ttPeak − lag time)), and area under the curve (or endogenous thrombin potential (ETP)). All patient samples were tested in triplicate: the analysed data is the average of the replicates. All clotting tests were performed in one laboratory and by one person to reduce variations.

### 2.5 Statistical analysis

The analysed dataset consisted of 31 patients with up to five TPE sessions each (total N = 131). Pre‐ and post‐exchange tests were made at each session and on each patient. Haemostatic responses were cleaned using clinical and statistical judgement, and levels of missing data were investigated and deemed acceptable in all cases. To examine the effect of TPE fluid type on haemostatic responses, two statistical approaches were used: (1) a linear mixed model with fluid type as the fixed effect, and (2) a canonical discriminant analysis. Four different fluid types were investigated; 5% Alb, 5% Alb + NS, Gelofusine + 5% Alb and Octaplas (either in isolation or mixed with other fluids). Twenty‐one haemostasis variables were analysed: FII, FV, FVII, FVIII, FIX, FX, FXI, FXII, Fibrinogen, PT, APTT, F1 + 2, TAT, PAI‐1, A2AP, plasminogen, lag‐time, peak‐thrombin height, ETP, ttPeak and VI. We were able to analyse all these variables with the models proposed, as a linear mixed model analyses one haemostatic variable at a time and canonical discriminant analysis is a dimension reduction technique especially robust at dealing with a large number of predictor variables. Tabachnick and Fidell^18^ recommend a sample size of at least twenty observations in the smallest group to ensure robustness of the analysis. All statistical analyses were carried out using the software package SAS Enterprise Guide © version 7.13 for Windows (2016 by SAS Institute Inc., Cary, North Caroline) which embeds Base SAS version 9.4.

### 2.6 Linear mixed modelling

The fixed component of the mixed model was set to *fluid type*. The random component was set to *patient ID* (to identify repeated measurements on the same patient over different exchange sessions) and *time interval* (to adjust for the time interval between a patient's consecutive exchange sessions, which forms a positive skew distribution that ranges from 1 to 30 days and with a median of 3 days). The response variable was taken as the *difference* between the post‐ and pre‐exchange measurements for each of the 21 haemostasis markers at each session and on each patient. For simplicity, our analysis assumed independence amongst haemostasis markers and separate linear mixed models were fitted to each marker.

### 2.7 Canonical discriminant analysis

A canonical linear discriminant analysis was performed using the *difference* between the post‐ and pre‐exchange measurements for each of the 21 haemostasis markers at each session as the predictors. *Fluid type* was used as the pre‐defined grouping variable of interest. The analysis identified the predictors that are most influential in discriminating between the fluid types. Canonical discriminant analysis is a multivariate statistical technique where independence of haemostatic parameters is not assumed and all parameters are considered simultaneously. As part of the discriminant analysis, we performed a multivariate one‐way analysis of variance (ANOVA).

## 3 RESULTS

### 3.1 Exploratory analysis

**FIGURE 1:**
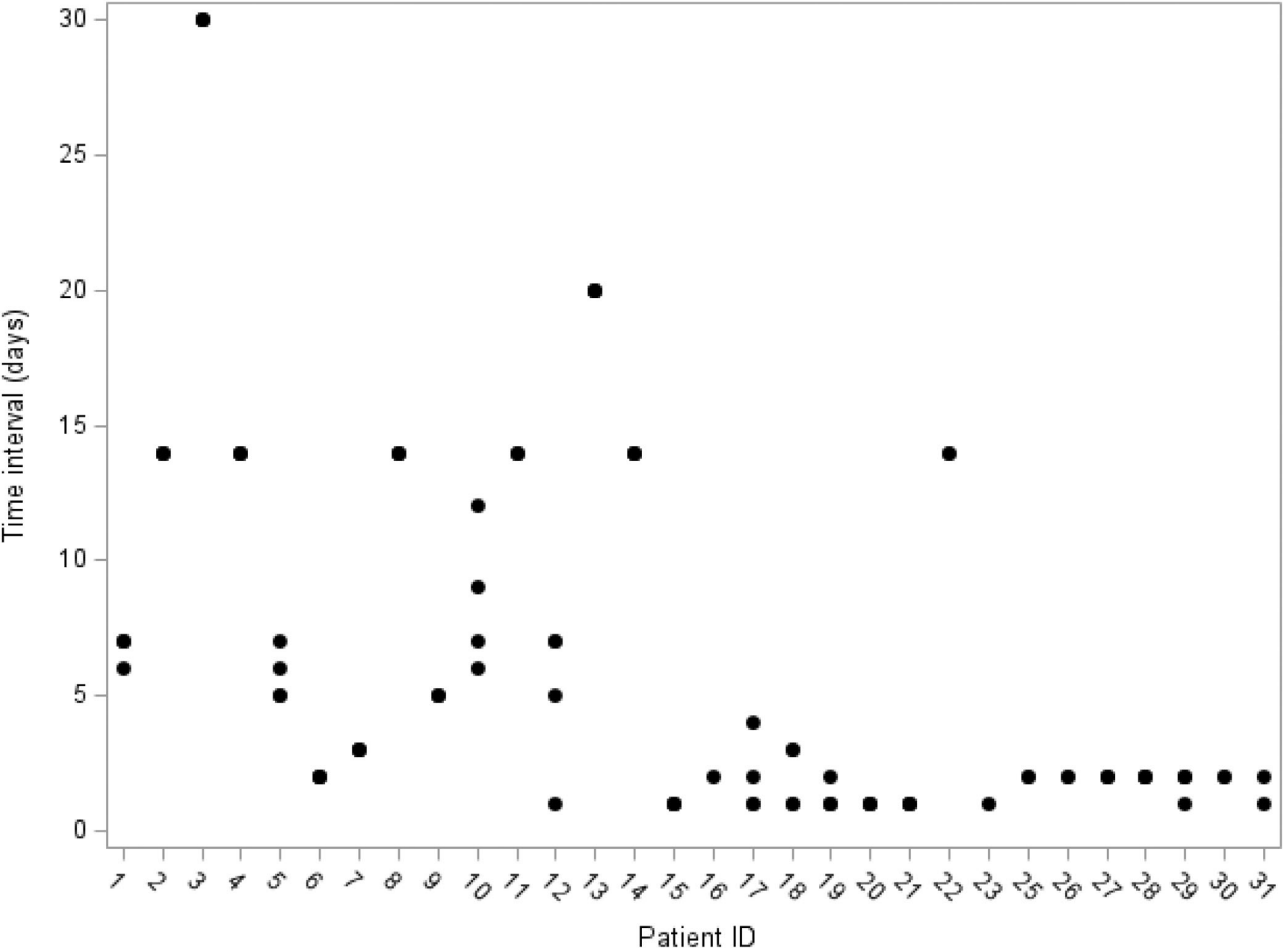
Scatter plot of patient ID vs the time interval (days) between a patient's two consecutive exchange sessions. The figure shows the scatter plot of patients vs the time interval between two consecutive exchange sessions. In the plot, each dot represents the time interval between two consecutive exchange sessions and identical time intervals for a given patient are overlaid. For instance, patient ID ‘2’ underwent five exchange sessions in total, all consecutive pairs with time interval equal to 14 days. Patient ID ‘10’ underwent five exchange sessions in total, resulting in four time intervals between consecutive pairs—all different, as shown in the figure. Patient ID ‘24’ does not appear in this figure as this patient underwent one exchange session only

Of the 31 patients in total, 20 (65%) underwent 5 TPE exchange sessions, 2 (6%) underwent 4 sessions, 6 (19%) experienced 3 sessions, 2 (6%) had 2 sessions and 1 patient (3%) underwent one exchange session only. This analysis did not attempt to determine how the number of TPE sessions affected haemostatic assay results. Characteristics of patients, reasons for needing TPE and the details of each fluid type per hospital are provided in Table 1. Of the 131 TPE sessions on 31 patients, 44 sessions (33%) used 5% Alb + NS (Hospital‐3 only), 43 sessions (33%) used 5% Alb + Gelofusine (Hospital‐2 only), 26 sessions (20%) used 5% Alb (Hospital‐1 and Hospital‐2), and 18 sessions (14%) used Octaplas either in isolation (n = 4) or mixed with other fluids (n = 14; Hospital‐1 and Hospital‐2). There were no complications related to TPE procedure. No patient had a personal or family history of bleeding disorders or relevant co‐morbidities which could potentially impact baseline factor levels. The scatter plot of patients vs the time interval between the patients' exchange sessions is described in Figure 1.

**TABLE 1:** Patient characteristics

Site	n (%) or mean (SD) or median (IQR)^a^		
	Hospital‐1 n = 7 patients	Hospital‐2 n = 15 patients	Hospital‐3 n = 9 patients
Age (years)^c^	37 (29‐55)	51 (46‐67)	57 (44.5‐71.5)
Gender			
Male	4 (57%)	12 (80%)	4 (44%)
Female	3 (43%)	3 (20%)	5 (56%)
Clinical Indication for performing TPE			
ABOi renal transplant	4 (57%)	2 (13%)	0
Vasculitis	0	4 (27%)	0
Hypercholesterolaemia	0	2 (13%)	0
Focal segmental glomerulosclerosis	1 (14%)	1 (7%)	0
Glutamic acid decarboxylase antibodies	2 (29%)	0	0
Other^b^	0	6 (40%)	9 (100%)
Number of TPE sessions	3.6 (0.97)	4.4 (1.12)	4.9 (0.33)
Replacement fluid type (pre and post sessions)			
5% Alb + normal saline	0	0	44 (100%)
5% Alb + Gelofusine	0	43 (69%)	0
5% Alb	17 (68%)	9 (15%)	0
5% Alb + Octaplas	7 (28%)	0	0
5% Alb + Octaplas + Gelofusine	0	7 (11%)	0
Octaplas®	1 (4%)	3 (5%)	0
Time interval between exchanges (days)^c^	2 (2–2)	2 (1‐14)	7 (5‐14)

*Notes*: Patients in hospital‐3 received the same fluid replacement throughout all their TPE sessions. In hospital‐1 and hospital‐2 Octaplas was added to the patient fluid replacement regimen if their fibrinogen level fell below 1.5 g/L on pre‐exchange bloods.

Abbreviations: ABOi, ABO incompatible; Alb, human albumin solution; IQR, interquartile range; TPE, therapeutic plasma exchange.

a n (%) in each category is given for categorical variables, mean (SD) is presented for continuous variables with an approximate normal distribution and median (IQR) is presented for continuous variables with a skewed distribution.

b Other includes primary biliary cirrhosis (n = 1), myelopathy (n = 2), myasthenia gravis (n = 1), hyperviscosity (n = 2), sarcoidosis (n = 1) and neuromyelitis optica (n = 1) or clinical indication not specified on the patient clinical data research form (n = 7).

^c^ Variable has a skewed distribution.

### 3.2 Effect of fluid type on haemostatic parameters using linear mixed modelling

Out of 21 markers analysed using linear mixed modelling, the main effects of *fluid type* were found to be statistically significant at the 5% level for 19 markers (*P* < 0.05 in all cases; see Table A1). The effects of fluid type were found to be non‐significant for PAI‐1 and TAT only. The five markers with the most significant fluid type effects were Fibrinogen, FXII, FII, peak height thrombin and FVII.

Table 2 shows the pair‐wise comparisons of fluid type least square means (comparisons are Bonferroni adjusted) that are statistically significant at the 5% level (“1”) or not (“0”). The mean Fibrinogen response when using 5% Alb compared with 5% Alb + normal saline (NS) was statistically significantly different, while the mean response when comparing 5% Alb with 5% Alb + Gelofusine was non‐significantly different. For most markers, a significant difference was found between Octaplas compared with any other fluid. In fact, the mean differences between 5% Alb + Gelofusine vs Octaplas were statistically significant (Bonferroni‐adjusted *P* < 0.05 in all cases) for all markers except thrombin generation parameters.

**TABLE 2:** Pair‐wise comparisons of least square means by haemostatic marker and fluid type pair

Fluid type pair/marker	A2AP	ETP	F1 + 2	FII	FIX	FV	FVII	FVIII	FX	FXI	FXII	Fib	PT	PH	PLG	Lag‐time	ttPeak	VI	APTT	Total
5% Alb vs Gelofusine + 5% Alb	0	0	0	0	0	0	0	0	0	0	0	0	1	0	0	0	0	0	0	1
5% Alb vs 5% Alb + NS	0	0	0	0	0	0	0	0	0	0	0	1	0	0	0	1	1	1	0	4
5% Alb + NS vs Gelofusine + 5% Alb	0	1	0	0	0	0	0	0	0	0	0	1	0	1	0	1	1	1	0	6
5% Alb + NS vs Octaplas	1	1	0	1	0	1	1	1	1	1	1	0	0	1	1	0	0	1	0	12
5% Alb vs Octaplas	1	1	1	1	1	1	1	0	1	1	1	0	0	1	1	0	0	0	0	12
Gelofusine + 5% Alb vs Octaplas	1	0	1	1	1	1	1	1	1	1	1	1	1	0	1	1	0	0	1	15
Total	3	3	2	3	2	3	3	2	3	3	3	3	2	3	3	3	2	3	1	50

*Notes*: All comparisons are Bonferroni adjusted. 1 = the difference of least square means is statistically significant at the 5% level (provided as shaded in the table); 0 = the difference is non‐significant.

Abbreviations: A2AP, alpha‐2‐antiplasmin; Alb, human albumin solution; APTT, activated partial thromboplastin time; ETP, endogenous thrombin potential; F1 + 2, prothrombin fragment 1 + 2; Fib, fibrinogen; PH, peak‐thrombin height; PLG, plasminogen; PT, prothrombin time; ttPeak, time to peak; VI, velocity index.

Table A2 shows the means, standard errors (SE) and 95% confidence limits (CL) as estimated by the linear mixed model. The data are the difference between the post‐ and pre‐exchange measurements at each session and on each patient. We are providing the means/SE/CL, as estimated by the model, as these (unlike raw mean/SD estimates) take account of the structure in the data, which is important in order to make inferences.

### 3.3 Haemostatic parameters to discriminate between fluid types

**FIGURE 2:**
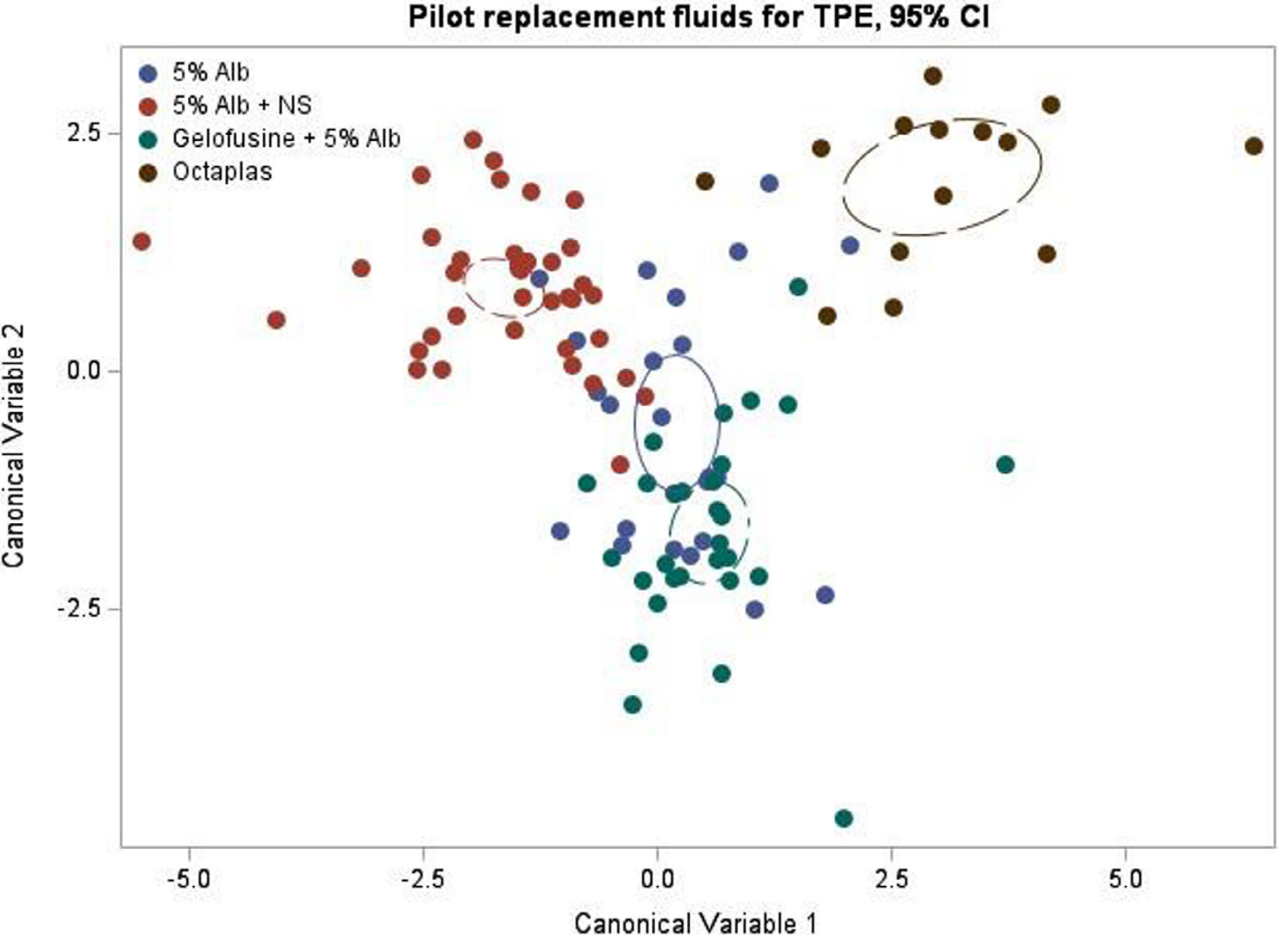
Plot of the first two canonical variables with 95% confidence bands around the group means: four‐group canonical discriminant analysis. Each dot in the figure is a transformation from a 21‐dimensional space (corresponding to the 21 haemostatic markers) into a two‐dimensional space (corresponding to the two canonical variables; CV) of the difference between post‐ and pre‐exchange measurements at each session. The transformation is achieved by specifying each CV as a linear combination of the 21 markers measured. The different colours correspond to the four replacement fluids (there were fewer sessions that used Octaplas) and the aim of the analysis is to find a CV‐transformation that best discriminates between the groups. By definition, CV1 has the highest discriminatory power, which is evident in the greater separation between the groups along the *x* axis in this figure. The analysis results tell us that CV1 is dominated by thrombin generation parameters (lag‐time and ttPeak), fibrinogen and FV as the top discriminating markers

We used canonical linear discriminant analysis as an alternative statistical approach to identify the haemostatic parameters that are most influential in discriminating between the four fluid type groups. A multivariate ANOVA on the hypothesis that the fluid type groups are equal indicated statistically significant differences between the groups (Wilks' lambda = 0.07; *F*_63,245.61_ = 5.50; *P* < 0.0001). Figure 2 shows the plot of the first two canonical variables (CV), which are directions in multivariate space that maximally discriminate the pre‐defined groups of interest specified in the data; in our study, the fluid types. Raw canonical coefficient estimates (not shown) for CV1 indicate that the top four discriminating markers driving the differences between fluid type groups were thrombin generation parameters (lag‐time and ttPeak), Fibrinogen and FV. Consistent with the linear mixed model analysis, the Octaplas group appears as a separate group when compared with the other fluid types (Figure 2).

A further canonical linear discriminant analysis of only three fluid type groups (excluding Octaplas) indicated statistically significant differences between the groups (Wilks' lambda = 0.18; *F*_42,138_ = 4.42; *P* < 0.0001). Raw canonical coefficient estimates (not shown) for CV1 in this analysis again identified thrombin generation parameters (lag‐time and ttPeak), Fibrinogen and FII as the top discriminating markers. Figure A1 shows the plot of the first two CV for this analysis. Consistent with our linear mixed model analysis, the groups that lie the furthest along the *x* axis in Figure A1 are 5% Alb + NS and 5% Alb + Gelofusine. In Table 2, this fluid type pair shows the largest number of significant differences (total of 6) amongst comparisons that do not involve Octaplas. Like the canonical discriminant analysis results, thrombin generation parameters (lag‐time, ttPeak) and Fibrinogen are statistically significant for most comparisons that do not involve Octaplas in Table 2 (rows “5% Alb vs Gelofusine + 5% Alb”, “5% Alb vs 5% Alb + NS” and “5% Alb + NS vs Gelofusine + 5% Alb”).

## 4 DISCUSSION

In this multicentre observational study, we comprehensively evaluated the effect of four different types of TPE fluid replacements on haemostasis, which were 5% Alb, 5% Alb + NS, Gelofusine + 5% Alb and Octaplas (either in isolation or mixed with other fluids). We enrolled 31 patients who received a total of 131 TPE sessions.

Consistent with previous studies, our data show that coagulation factors pre and post exchanges reduce with all types of fluids.^9^, ^19^, ^20^ However, unlike other studies, in our assessment we also evaluated the effect of different TPE fluid types on in vitro thrombin generation and as such we were able to show that the four most discriminating haemostatic markers, driving the differences between fluid types were lag‐time, ttPeak (thrombin generation), Fibrinogen and FV. Thrombin generation test is a physiological global clotting test, which due to lack of standardisation has not yet been introduced into routine clinical practice and thus, it is currently used as a research tool. In this study the impact of TPE fluid was mainly seen in the initiation and propagation phases of thrombin generation, with less effect on the ETP. Based on results of other haemostatic tests, we believe that this can be explained due to changes related to fibrinogen levels^21^ as this variable was the most discriminatory variable compared to other factors that drive the ETP (like FVIII). Fibrinogen is a key component of haemostasis and other studies have shown that its levels reduce significantly with all fluid types assessed here^9^, ^10^, ^19^, ^20^ making it a good marker for assessing an important part of haemostasis. Indeed, all hospitals that took part in this study recommend in their local protocols that fibrinogen levels be measured before and after TPE to determine the immediate haemostatic status of patients following TPE procedures. The British Society for Haematology (BSH) guideline on the clinical use of apheresis procedures recommends monitoring fibrinogen levels carefully during the course of plasma exchange.^22^ The most recent American Society for Apheresis (ASFA) guidelines recommend monitoring of fibrinogen levels especially where treatments are performed daily^23^ with ASFA “choosing wisely campaign” advising against routine monitoring of coagulation tests during a course of TPE unless the procedure is performed daily.^24^ In our study, all three sites included the measurement of fibrinogen pre‐TPE in their standard protocols, but only one site would measure it post‐TPE for patients at high risk of bleeding only.

In both our analyses the difference between Octaplas and other fluid types was statistically significant, which is not surprising when we consider the difference in content between Octaplas and other fluids (the former being a plasma product). Of the non‐Octaplas fluids, the use of NS as a replacement fluid for TPE mixed with 5% Alb had a distinctive effect on thrombin generation and fibrinogen responses (the most discriminating CV1 markers) compared to other non‐plasma fluid types, as shown in Figure 2, where the NS group is well separated along the x axis and lies on the opposite side of zero relative to other fluid types. This is an important finding because it does suggest that NS could impair the haemostatic balance in these patients and larger longitudinal studies are needed to evaluate if this haemostatic effect has an impact on clinical outcomes. From the recent systematic scoping review that we completed (accepted for publication to Journal of Clinical Apheresis^25^), we identified no studies where NS had been used as a stand‐alone fluid replacement for TPE, but we did identify three studies (two randomised control trials^26^, ^27^ and one prospective cohort^28^) where NS was used as a fluid mix with Alb with no comparator fluid replacement arm. TPE in all three studies was done for different underlying clinical indications with small sample size numbers (lupus nephritis, n = 40^26^; rheumatoid arthritis, n = 10^27^; multiple sclerosis, n = 18^28^). Outcome measures used to determine efficacy of fluid replacement were disease specific and none of the three studies showed any benefit of TPE over standard treatment. Randomised control trials are needed to compare the cost‐effectiveness of different TPE fluids,^29^ and in particular NS.

## 5 STRENGTHS AND LIMITATIONS

This is the first multi‐centre study providing a comprehensive haemostatic assessment of more than two different replacement fluid types pre and post TPE. However, there are limitations to this study such as: being an observational study, and the population of patients being intrinsically heterogeneous with clinical indication for TPE and duration between TPE sessions being different at each of the three hospital sites, although we account for these in our modelling.

The multivariate nature of canonical discriminant analysis and multivariate ANOVA—where all haemostatic markers are considered simultaneously, is appropriate when variables are correlated (Figure A2) and independence between markers is difficult to justify as in our linear mixed modelling approach. However, neither canonical discriminant analysis nor multivariate ANOVA takes account of structure in the data, such as repeated measurements. One limitation of our canonical discriminant analysis is the small sample size in the Octaplas group (n = 18; Table 1), which calls for the use of complementary statistical techniques to take account of data features and limitation in methods; we found agreement in the results produced by the alternative techniques.

## 6 CONCLUSION

Thrombin generation and fibrinogen were the best markers for discriminating between different TPE fluid types. Further studies are needed to evaluate the relationship between these markers and clinical efficacy/safety of TPE procedures, with the view to using them as potential outcome measures for study evaluation or clinical practice. The effect of Octaplas on haemostasis was significantly different compared with other fluid types, while the use of NS in combination with other TPE fluid, had a distinctive impact on haemostasis (vs other non‐Octaplas fluid types), in particular on thrombin generation and fibrinogen responses. Future longitudinal studies are needed to evaluate if this effect has an impact on clinical outcomes, and randomised control trials should compare the cost‐effectiveness of different TPE fluids.

## CONFLICTS OF INTEREST

The authors declare no conflicts of interest.

## Supporting information


**Appendix S1** Supporting information

**Appendix S2** Supporting information

## Data Availability

The data that supports the findings of this study are available in the supplementary material of this article
