# Supplementary material for: Effect on haemostasis of different replacement fluids during therapeutic plasma exchange—A comparative multicentre observational study
Source: J Clin Apher. 2022 Aug 24;37(6):534–43. doi: 10.1002/jca.22008 (PMC10087465; doi:10.1002/jca.22008)
Supplement: Supplementary file 1 — Appendix S1 Supporting information [file JCA-37-534-s002.docx]

**SUPPLEMENTARY INFORMATION**

**Table A1.** Significance tests for the fluid type fixed effect, independently obtained for each marker. The p values have been ordered from the most significant (at the top) to the least significant (at the bottom).

| **Marker** | **Num DF*** | **Den DF*** | **F Value** | **p value** |
| --- | --- | --- | --- | --- |
| Fibrinogen | 3 | 83 | 17.69 | 0.000000006 |
| FXII | 3 | 83 | 16.94 | 0.000000011 |
| FII | 3 | 83 | 15.54 | 0.000000041 |
| Peak thrombin height | 3 | 68 | 14.20 | 0.000000278 |
| FVII | 3 | 83 | 12.13 | 0.000001168 |
| FX | 3 | 83 | 11.63 | 0.000001947 |
| FXI | 3 | 83 | 9.59 | 0.000016711 |
| FV | 3 | 83 | 9.56 | 0.000017243 |
| ETP | 3 | 68 | 9.52 | 0.000024699 |
| VI | 3 | 68 | 9.43 | 0.000027262 |
| Lag-time | 3 | 65 | 9.14 | 0.000039321 |
| Plasminogen | 3 | 83 | 8.04 | 0.000090855 |
| ttPeak | 3 | 65 | 7.90 | 0.000143239 |
| PT | 3 | 84 | 6.14 | 0.000792822 |
| F1+2 | 3 | 84 | 5.58 | 0.001535932 |
| FVIII | 3 | 83 | 5.52 | 0.001653769 |
| FIX | 3 | 83 | 4.18 | 0.008306817 |
| A2AP | 3 | 84 | 4.03 | 0.009967483 |
| APTT | 3 | 84 | 2.89 | 0.040124316 |
| PAI-1 | 3 | 84 | 2.25 | 0.088111756 |
| TAT | 3 | 84 | 1.26 | 0.292072351 |

* DF = degrees of freedom

ETP, endogenous thrombin potential; VI, velocity index; ttPeak, time to peak; PT, prothrombin time; F1+2, prothrombin fragment 1+2; A2AP, alpha-2-antiplasmin; APTT, activated partial thromboplastin time; PAI-1, plasminogen activator inhibitor-1; TAT, thrombin antithrombin complex

Some patients had pre or post measurements missing for thrombin generation parameters due to “flat curves” generated on thrombin generation analysis. A comment has been added under methods.

**Table A2.** Least square means and standard errors of the linear mixed model fixed effects. The data are the difference between the post- and pre- exchange measurements at each session and on each patient.

| **Haemostatic parameter** | **Fluid type** | **Mean** | **Standard Error** | **95% lower confidence limit** | **95% upper confidence limit** |
| --- | --- | --- | --- | --- | --- |
| A2AP | 5% HAS | -53.15 | 5.42 | -63.93 | -42.38 |
| A2AP | 5% HAS + NS | -55.15 | 4.71 | -64.51 | -45.80 |
| A2AP | Gelofusine + 5% HAS | -49.92 | 4.46 | -58.78 | -41.05 |
| A2AP | Octaplas | -32.18 | 6.10 | -44.30 | -20.06 |
| ETP | 5% HAS | -139.06 | 80.12 | -298.94 | 20.82 |
| ETP | 5% HAS + NS | -228.84 | 75.61 | -379.72 | -77.96 |
| ETP | Gelofusine + 5% HAS | 136.20 | 71.80 | -7.07 | 279.47 |
| ETP | Octaplas | 309.90 | 90.18 | 129.96 | 489.84 |
| F1+2 | 5% HAS | -87.57 | 66.00 | -218.81 | 43.68 |
| F1+2 | 5% HAS + NS | 26.24 | 53.21 | -79.57 | 132.06 |
| F1+2 | Gelofusine + 5% HAS | -39.53 | 52.42 | -143.77 | 64.72 |
| F1+2 | Octaplas | 223.55 | 77.78 | 68.89 | 378.22 |
| FII | 5% HAS | -47.33 | 4.40 | -56.07 | -38.59 |
| FII | 5% HAS + NS | -38.60 | 3.64 | -45.83 | -31.36 |
| FII | Gelofusine + 5% HAS | -49.34 | 3.55 | -56.41 | -42.28 |
| FII | Octaplas | -16.54 | 5.10 | -26.68 | -6.40 |
| FIX | 5% HAS | -54.11 | 8.35 | -70.72 | -37.50 |
| FIX | 5% HAS + NS | -48.94 | 8.03 | -64.90 | -32.97 |
| FIX | Gelofusine + 5% HAS | -56.40 | 7.20 | -70.72 | -42.09 |
| FIX | Octaplas | -25.43 | 8.62 | -42.58 | -8.28 |
| FV | 5% HAS | -50.18 | 7.36 | -64.82 | -35.55 |
| FV | 5% HAS + NS | -64.04 | 7.59 | -79.14 | -48.95 |
| FV | Gelofusine + 5% HAS | -58.39 | 6.48 | -71.28 | -45.51 |
| FV | Octaplas | -22.29 | 7.41 | -37.03 | -7.55 |
| FVII | 5% HAS | -46.99 | 3.93 | -54.82 | -39.16 |
| FVII | 5% HAS + NS | -48.30 | 3.16 | -54.59 | -42.01 |
| FVII | Gelofusine + 5% HAS | -39.86 | 3.14 | -46.11 | -33.62 |
| FVII | Octaplas | -16.78 | 4.64 | -26.02 | -7.55 |
| FVIII | 5% HAS | -82.26 | 13.78 | -109.66 | -54.86 |
| FVIII | 5% HAS + NS | -99.64 | 12.98 | -125.45 | -73.82 |
| FVIII | Gelofusine + 5% HAS | -101.41 | 11.80 | -124.88 | -77.94 |
| FVIII | Octaplas | -38.61 | 14.43 | -67.30 | -9.92 |
| FX | 5% HAS | -42.76 | 4.43 | -51.57 | -33.95 |
| FX | 5% HAS + NS | -47.94 | 3.61 | -55.12 | -40.77 |
| FX | Gelofusine + 5% HAS | -43.72 | 3.55 | -50.77 | -36.66 |
| FX | Octaplas | -15.74 | 5.18 | -26.05 | -5.43 |
| FXI | 5% HAS | -49.82 | 7.12 | -63.97 | -35.66 |
| FXI | 5% HAS + NS | -57.87 | 6.95 | -71.69 | -44.05 |
| FXI | Gelofusine + 5% HAS | -53.02 | 6.18 | -65.32 | -40.72 |
| FXI | Octaplas | -18.51 | 7.44 | -33.30 | -3.71 |
| FXII | 5% HAS | -44.90 | 5.66 | -56.16 | -33.63 |
| FXII | 5% HAS + NS | -46.58 | 5.38 | -57.28 | -35.87 |
| FXII | Gelofusine + 5% HAS | -40.21 | 4.86 | -49.89 | -30.54 |
| FXII | Octaplas | -5.09 | 6.01 | -17.05 | 6.87 |
| Fibrinogen | 5% HAS | -1.13 | 0.21 | -1.55 | -0.71 |
| Fibrinogen | 5% HAS + NS | -0.12 | 0.19 | -0.50 | 0.27 |
| Fibrinogen | Gelofusine + 5% HAS | -1.65 | 0.18 | -2.01 | -1.28 |
| Fibrinogen | Octaplas | -0.82 | 0.23 | -1.28 | -0.36 |
| PAI-1 | 5% HAS | -6.62 | 4.21 | -15.00 | 1.75 |
| PAI-1 | 5% HAS + NS | 2.10 | 3.21 | -4.30 | 8.49 |
| PAI-1 | Gelofusine + 5% HAS | -8.01 | 3.23 | -14.43 | -1.59 |
| PAI-1 | Octaplas | 0.61 | 5.09 | -9.51 | 10.73 |
| PT | 5% HAS | 0.92 | 0.99 | -1.04 | 2.88 |
| PT | 5% HAS + NS | 3.51 | 0.90 | 1.71 | 5.30 |
| PT | Gelofusine + 5% HAS | 5.18 | 0.83 | 3.52 | 6.83 |
| PT | Octaplas | 0.76 | 1.06 | -1.35 | 2.88 |
| Peak thrombin height | 5% HAS | -48.63 | 18.31 | -85.18 | -12.09 |
| Peak thrombin height | 5% HAS + NS | -109.38 | 17.26 | -143.82 | -74.94 |
| Peak thrombin height | Gelofusine + 5% HAS | 4.93 | 16.41 | -27.81 | 37.67 |
| Peak thrombin height | Octaplas | 53.20 | 20.64 | 12.01 | 94.40 |
| Plasminogen | 5% HAS | -45.37 | 5.71 | -56.73 | -34.02 |
| Plasminogen | 5% HAS + NS | -55.23 | 4.88 | -64.94 | -45.51 |
| Plasminogen | Gelofusine + 5% HAS | -43.73 | 4.61 | -52.91 | -34.56 |
| Plasminogen | Octaplas | -19.57 | 6.26 | -32.02 | -7.12 |
| TAT | 5% HAS | -0.78 | 0.64 | -2.06 | 0.50 |
| TAT | 5% HAS + NS | -1.60 | 0.58 | -2.76 | -0.44 |
| TAT | Gelofusine + 5% HAS | -2.22 | 0.54 | -3.30 | -1.14 |
| TAT | Octaplas | -2.24 | 0.70 | -3.63 | -0.85 |
| Lag-time | 5% HAS | -1.32 | 0.23 | -1.78 | -0.85 |
| Lag-time | 5% HAS + NS | -0.40 | 0.22 | -0.85 | 0.04 |
| Lag-time | Gelofusine + 5% HAS | -1.60 | 0.21 | -2.02 | -1.19 |
| Lag-time | Octaplas | -0.79 | 0.25 | -1.30 | -0.29 |
| ttPeak | 5% HAS | -2.58 | 0.47 | -3.51 | -1.65 |
| ttPeak | 5% HAS + NS | -0.46 | 0.44 | -1.33 | 0.41 |
| ttPeak | Gelofusine + 5% HAS | -2.81 | 0.41 | -3.64 | -1.99 |
| ttPeak | Octaplas | -1.79 | 0.51 | -2.81 | -0.77 |
| VI | 5% HAS | -0.78 | 9.31 | -19.37 | 17.81 |
| VI | 5% HAS + NS | -41.38 | 8.61 | -58.57 | -24.19 |
| VI | Gelofusine + 5% HAS | 11.30 | 8.13 | -4.93 | 27.52 |
| VI | Octaplas | 21.49 | 10.43 | 0.68 | 42.30 |
| APTT | 5% HAS | 7.22 | 2.76 | 1.73 | 12.71 |
| APTT | 5% HAS + NS | 5.61 | 2.59 | 0.45 | 10.77 |
| APTT | Gelofusine + 5% HAS | 10.15 | 2.36 | 5.46 | 14.84 |
| APTT | Octaplas | 0.77 | 2.90 | -4.99 | 6.53 |

**Figure A1.** Plot of the first two canonical variables with 95% confidence bands around the group means: three-group canonical discriminant analysis.

**
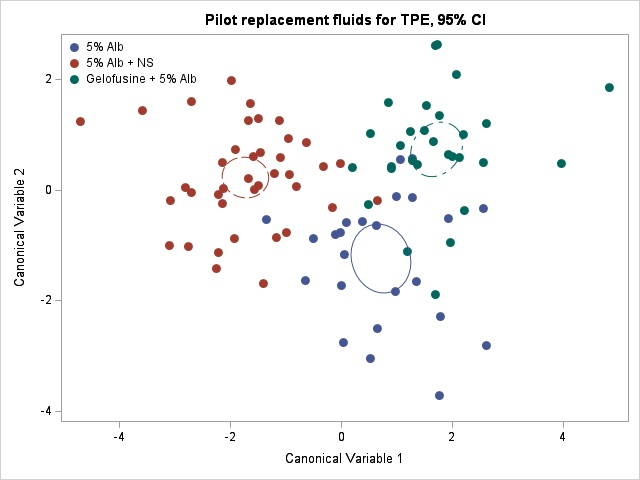
**

*Each dot in the figure is a transformation from a 21-dimensional space (corresponding to the 21 haemostatic markers) into a two-dimensional space (corresponding to the two canonical variables; CV) of the difference between post- and pre-exchange measurements at each session. The transformation is achieved by specifying each CV as a linear combination of the 21 markers measured. The different colours correspond to the three replacement fluids and the aim of the analysis is to find a CV-transformation that best discriminates between the groups. By definition, CV1 has the highest discriminatory power, which is evident in the greater separation between the groups along the x axis in* ***Figure A1****. The analysis results tell us that CV1 is dominated by thrombin generation parameters (lag-time and ttPeak), Fibrinogen and FII as the top discriminating markers.*

**Figure A2.** Scatter plots of pairs of coagulation factor analytes, coloured by fluid type.


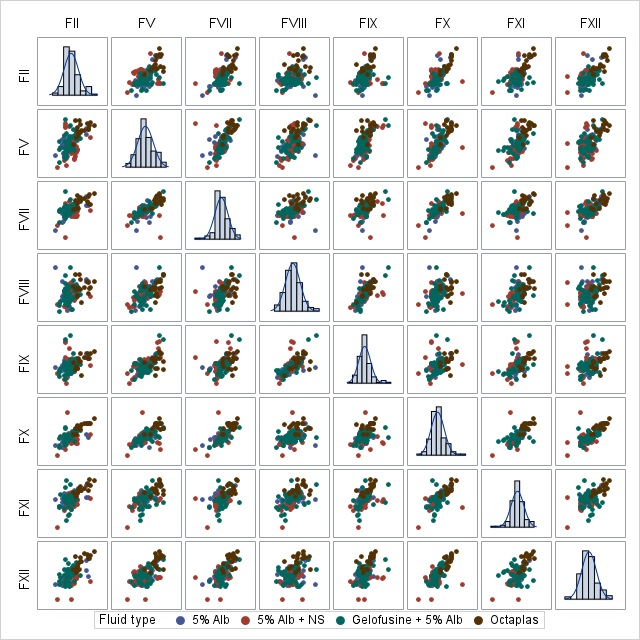


*The off-diagonal panels show the difference between the post- and pre-exchange measurements for pairs of F, factors, i.e. each dot in the plot is the post-pre difference. The diagonal of the matrix shows the distribution of each analyte, superimposed by a normal curve, which gives an indication of normally distributed analyte data. There is a noticeable correlation between factors FII to FXII. As variables are correlated it is appropriate to perform canonical discriminant analysis and multivariate ANOVA, where all haemostatic markers are considered simultaneously.*
